# A Rare Mutation in The *APOB* Gene Associated with Neurological Manifestations in Familial Hypobetalipoproteinemia

**DOI:** 10.3390/ijms21041439

**Published:** 2020-02-20

**Authors:** Joanna Musialik, Anna Boguszewska-Chachulska, Dorota Pojda-Wilczek, Agnieszka Gorzkowska, Robert Szymańczak, Magdalena Kania, Agata Kujawa-Szewieczek, Małgorzata Wojcieszyn, Marek Hartleb, Andrzej Więcek

**Affiliations:** 1 Department of Nephrology, Transplantation and Internal Medicine, Medical University of Silesia in Katowice, 40-055 Katowice, Poland; agata.szewieczek@gmail.com (A.K.-S.); awiecek@sum.edu.pl (A.W.); 2 Genomed SA, 02-971 Warsaw, Poland; annab-ch@genomed.pl (A.B.-C.); robert.szymanczak@genomed.pl (R.S.); magdalena.kania@genomed.pl (M.K.); 3 Department of Ophthalmology, Medical University of Silesia in Katowice, 40-055 Katowice, Poland; pojda-wilczek@wp.pl; 4 Department of Neurology, Department of Neurorehabilitation, Medical University of Silesia in Katowice, 40-055 Katowice, Poland; a_gorzkowska@wp.pl; 5 Department of Gastroenterology, II John Paul Pediatric Center, 41-200 Sosnowiec, Poland; mwojcieszyn@op.pl; 6 Department of Gastroenterology and Hepatology, Medical University of Silesia in Katowice, 40-055 Katowice, Poland; mhartleb@sum.edu.pl

**Keywords:** familial hypobetalipoproteinemia, nonalcoholic fatty liver disease, liver steatosis, *APOB* mutation

## Abstract

Clinical phenotypes of familial hypobetalipoproteinemia (FHBL) are related to a number of defective apolipoprotein B (*APOB*) alleles. Fatty liver disease is a typical manifestation, but serious neurological symptoms can appear. In this study, genetic analysis of the *APOB* gene and ophthalmological diagnostics were performed for family members with FHBL. Five relatives with FHBL, including a proband who developed neurological disorders, were examined. A sequencing analysis of the whole coding region of the *APOB* gene, including flanking intronic regions, was performed using the next-generation sequencing (NGS) method. Electrophysiological ophthalmological examinations were also done. In the proband and his affected relatives, NGS identified the presence of the pathogenic, rare heterozygous splicing variant c.3696+1G>T. Two known heterozygous missense variants—c.2188G>A, p.(Val730Ile) and c.8353A>C, p.(Asn2785His)—in the *APOB* gene were also detected. In all patients, many ophthalmologic abnormalities in electrophysiological tests were also found. The identified splicing variant c.3696+1G>T can be associated with observed autosomal, dominant FHBL with coexisting neurological symptoms, and both identified missense variants could be excluded as the main cause of observed clinical signs, according to mutation databases and the literature. Electroretinography examination is a sensitive method for the detection of early neuropathy and should therefore be recommended for the care of patients with FHBL.

## 1. Introduction

Nonalcoholic fatty liver disease (NAFLD) is now the most frequent chronic liver disease worldwide. The median prevalence of this disease, closely associated with obesity and metabolic syndrome, is 20% worldwide and 25–26% in Europe [1]. However, in some cases, classical NAFLD must be distinguished from genetically driven secondary NAFLD not directly associated with insulin-resistance. One such example is familial hypobetalipoproteinemia (FHBL) [2,3,4]. 

FHBL is a monogenic codominant disorder that may be due to loss-of-function mutations in the apolipoprotein B (*APOB*) or, less frequently, the *PCSK9* or *ANGPTL3* genes [5]. The disease is characterized by reduced serum levels of total cholesterol (TC), low-density lipoproteins (LDL), and apolipoprotein B (apoB). Triglycerides (TG) are entrapped in hepatocytes, and reduced access to cholesterol is responsible for decreased synthesis of bile acids, resulting in malabsorption of fat-soluble vitamins. Patients with FHBL can be homozygotes or heterozygotes, and the clinical phenotypes related to the number of defective *APOB* alleles. Homozygotic FHBL has profound clinical consequences if left untreated. Patients can develop atypical retinitis pigmentosa, severe ataxia, dysarthria, and absent reflexes, leading to dramatic neurological functional impairment and reduced lifespan. Other clinical manifestations include malabsorption syndrome and fatty liver disease [6]. The mechanism of fat accumulation within the liver development is decreased hepatic secretion of apoB resulting in reduced triglyceride export from the liver. Therefore, hepatic steatosis with mild elevation of liver enzymes is the main clinical manifestation of heterozygous FHBL [7]. Heterozygotes often have no clinical manifestations other than very low serum apoB-containing lipoprotein levels, which may actually confer cardiovascular protection, but an undetermined proportion of subjects may also suffer from liver and systemic metabolic consequences of this disease [8,9,10]. The preliminary differential diagnosis between homo- and heterozygotic patients is based on an evaluation of TC, LDL, and TG levels. In homozygous and heterozygous individuals, the TC and LDL levels that confirm the diagnosis are <80 and <20 mg/dL and <120 and <80 mg/dL, respectively. In the homozygous form of FHBL, the TG level is very low, while in the heterozygous form of this disease, it is usually normal [7]. 

Up to now, over 140 mutations in *APOB*, *PCSK9*, *ANGPTL3*, *MTTP*, and *SAR1* genes have been identified, of which the majority are point mutations resulting in either splicing errors or premature truncations [8,11,12,13]. The definitive “gold standard” diagnostic test for FHBL and the clinically indistinguishable autosomal recessive disorder abetalipoproteinemia (ABL) would, therefore, be a next-generation sequencing (NGS) panel covering all these genes.

In this study, we report a medical history and molecular investigation conducted in one family presenting clinical features of heterozygous FHBL carrying a rare pathogenic variant connected with strong neurological manifestations.

### Family Description 

**Figure 1:**
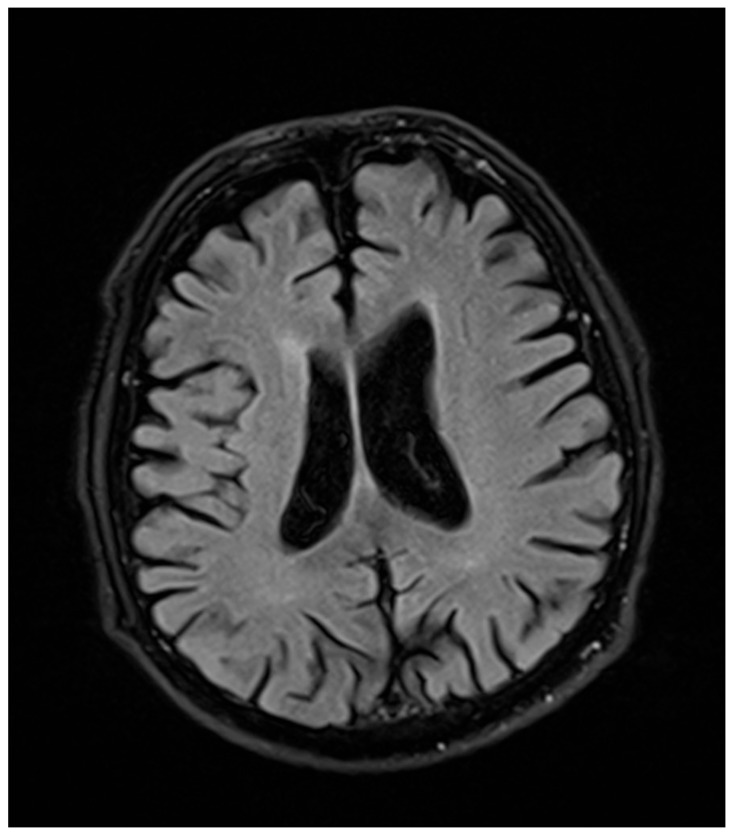
Magnetic resonance (MR) examination of the head performed two years before patient’s death. The cortical and subcortical atrophy is marked.

**Figure 2:**
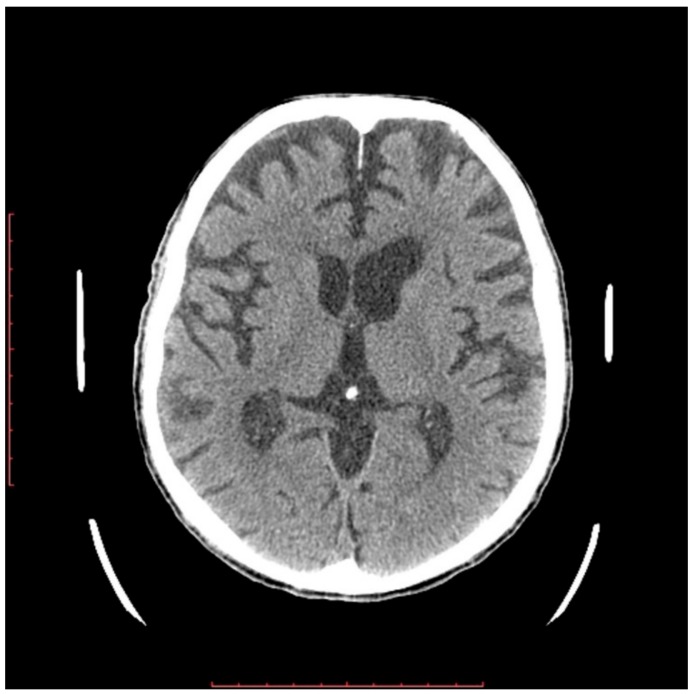
Computed tomography (CT) examination made before patient’s death. Progression of frontal lobe and subcortical atrophy with secondary ventricular dilatation are shown.

In a 76-year-old male proband, a liver biopsy performed at the age of 64 disclosed nonalcoholic steatohepatitis (NASH) with periportal fibrosis. At the same time, laboratory abnormalities typical of heterozygous FHBL were found. Because of the asymptomatic course, no treatment was implemented. At the age of 71, he noted slurred speech, bradykinesia, and upper-limb tremor. Neurological examination at that time revealed hypokinetic dysarthria, dysphagia (Swallowing Rating Scale of 6), bradykinesia (especially in upper limbs), upper-left-limb apraxia and adiadochokinesia, postural tremor of upper limbs, asymmetrical knee tendon reflexes, and reduced Achilles tendon reflexes. After three years of follow-up, he showed significant progression of dysarthria, dysphagia, and left-limb ataxia after three years of follow-up. Additionally, he had excessive daytime sleepiness, he stopped playing bridge, and complained of “weakness” in his left upper limb, dizziness, and postural instability. Due to walking difficulties he used a wheelchair. At the age of 76, a neurological examination revealed cognitive impairment, bulbar palsy, combined pyramidal–extrapyramidal syndrome, bilateral hand muscle atrophy, and bilateral positive Babinski signs. A cranial computed tomography (CT) scan showed cortico–subcortical atrophy. Five years after development of clinically overt symptoms, the patient died. In Figure 1 and Figure 2, the progression of cortical and subcortical atrophy is shown.

**Figure 3:**
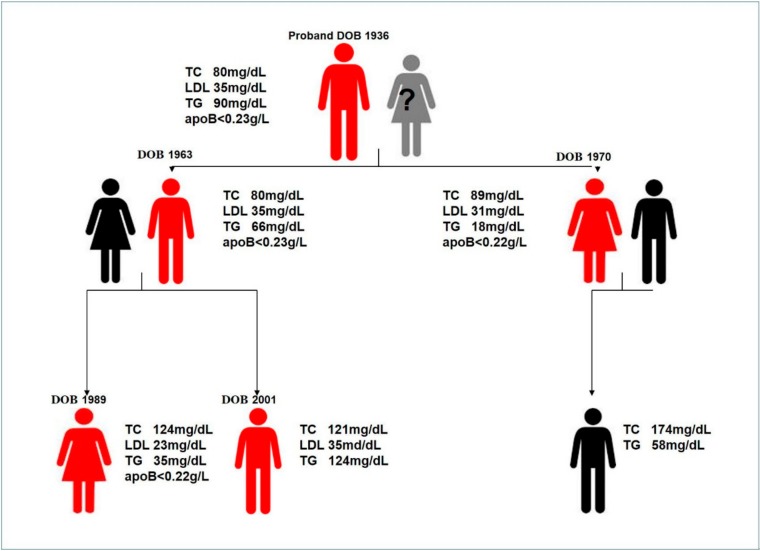
Family presentation with corresponding individual serum levels of total cholesterol (TC), low-density lipoprotein (LDL), triglycerides (TG), and apolipoprotein B (apoB). The red color represents affected family members, black represents healthy members, and grey represents a lack of information. DOB—date of birth.

When the neurological symptoms of the proband deteriorated, complete diagnostics were performed on members of his family: the children (son aged 50 years and daughter aged 44 years) and three grandchildren (granddaughter aged 26 years and two grandsons aged 15 and 13 years). Complete diagnostics in the Department of Nephrology, Transplantation and Internal Medicine and the Department of Gastroenterology and Hepatology were performed when the neurological symptoms of the proband increased. Low TC, LDL, and apoB serum levels were found in both children, and additionally, NAFLD was diagnosed from the liver biopsy, showing steatotic involvement of 40% (daughter) and 70% (son) of hepatocytes. In the granddaughter, ultrasound signs of liver steatosis and biochemical alterations typical for FHLB were found. In the ultrasound examination on the older grandson, the liver had normal echogenic structure and was not enlarged. In all adult subjects, including the proband, the serum levels of fat-soluble vitamins were normal, but near the low border of reference values. In Figure 3, the family presentation is given. Because of neurological manifestations in the heterozygous proband, we decided to carry out genetic diagnostics for all family members.

## 2. Results

### 2.1. Genetic Testing

**Figure 4:**
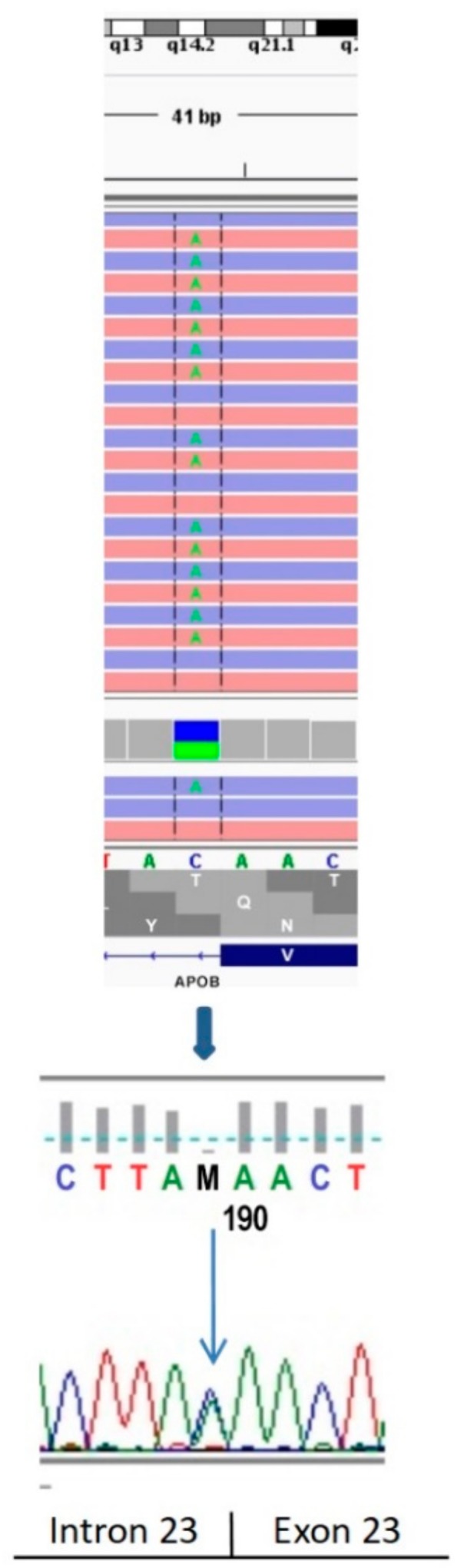
Next-generation sequencing (NGS)-based identification and Sanger sequencing confirmation of the c.3696+1G>T mutation.

In the proband, the NGS analysis identified the presence of a rare, heterozygous splicing variant c.3696+1G>T, changing the first base of intron 23 of the *APOB* gene (*APOB* genotype according to the Human Genome Variation Society (HGVS): NM_000384.2:c.[3696+1G>T];[=]). Sequencing results are presented in Figure 4.

A bioinformatics analysis performed using the Alamut software v.2.4 (Interactive Biosoftware, Rouen, France) indicated that the identified c.3696+1G>T variant is localized in the invariant splicing donor site and probably impairs *APOB* splicing. 

Two known heterozygous missense variants in the *APOB* gene—c.2188G>A, p.Val730Ile (rs12691202) in exon 15 and c.8353A>C, p.Asn2785His (rs2163204) in exon 26—were also detected. 

**Figure 5:**
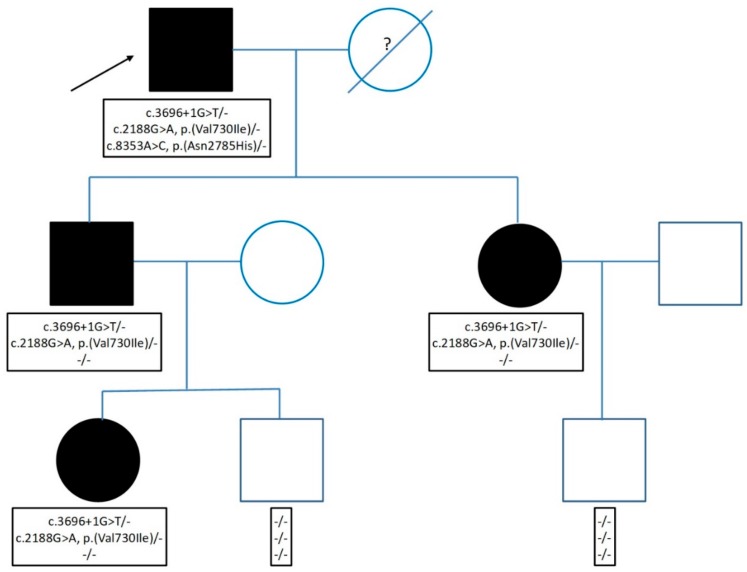
Family pedigree indicating the *APOB* variants identified for the proband and his family members. The proband is indicated with an arrow, circles correspond to female and squares to male family members. Filled symbols indicate affected family members and empty symbols indicate healthy members. The question mark represents a lack of information (death before the analysis).

An analysis performed in the proband’s family using targeted Sanger sequencing of the three variants identified revealed that the splicing variant (c.3696+1G>T) was found in the affected proband’s relatives (Figure 5), so it co-segregated with the disease symptoms.

### 2.2. Neurological Examination

In the neurological examination, the son of the proband had a mild hand tremor and the granddaughter had a mild head tremor. No other pathology was found in the tests performed. 

### 2.3. Ophthalmological Examination 

In all patients, normal corrected visual acuity (1.0 decimal) was found and intraocular pressure was lower than 19 mmHg. An abnormal visual field with numerous scotomas was found in the proband only. 

Optical coherence tomography (OCT) was not performed in the proband due to technical reasons. In all other family members, normal macular thickness was found. The ganglion cell layer was normal in all male patients (average thickness of 78 µm) and lower than normal in female patients (average thickness of 73 µm in younger patient and 70 µm in the older one). 

**Figure 6:**
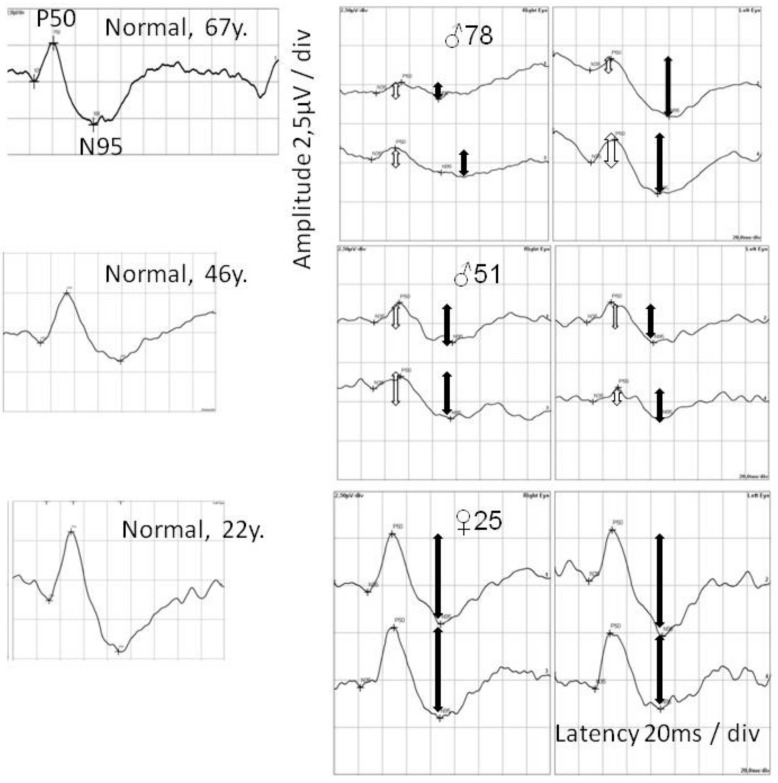
Pattern electroretinography (PERG) in the patients from three generations (proband, his son, and granddaughter—right figures) in comparison with results obtained from healthy men (left figures). Lower P50 wave amplitude in the proband and his son is marked with an empty arrow. Reduced N95 wave amplitude in all patients is marked with a filled arrow.

**Figure 7:**
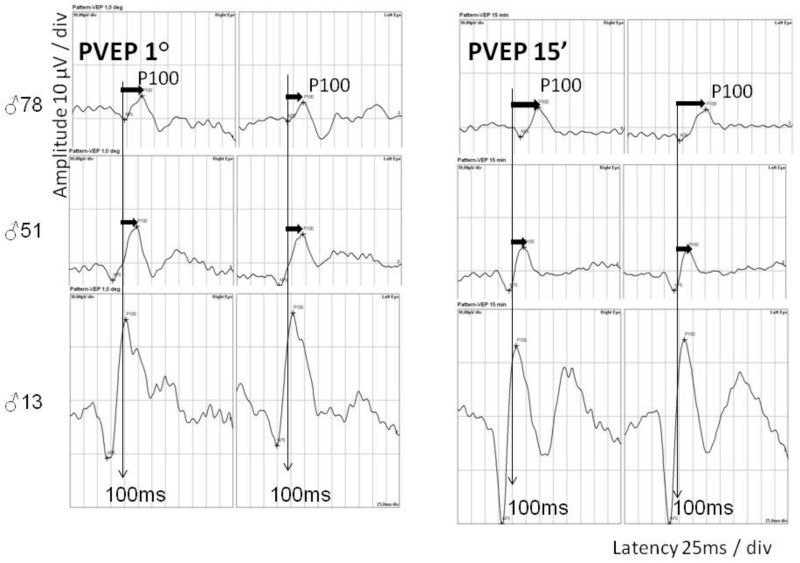
Pattern visual evoked potentials (PVEP) in patients from three generations. P100 wave amplitude within normal age range; P100 wave latency delayed (filled arrow).

In the flash full-field electroretinography (FERG) examination, a delay in implicit time (1–5 ms above upper limit) and abnormally low amplitudes of a and b waves in both the scotopic and photopic conditions (60–80% of the normal mean amplitude in the proband and his children and 90% in his granddaughter) were found. In the pattern electroretinography (PERG) examination, reduced amplitude of P50 and N95 was noted: 30–50% of normal mean amplitude in the proband and his children and 90% in his granddaughter (Figure 6). In the pattern visual evoked potentials (PVEP) examination, prolonged P100 latency was noticed in the proband and his children (Figure 7). In the grandchildren, P100 latency was close to the upper limit. The prolongation of P100 wave latency increased with the age of the patients.

The results of the ophthalmological examinations of healthy boys were normal. 

## 3. Discussion

The spectrum of clinical manifestations of FHBL is related to the number of mutated *APOB* alleles, and it is believed that most severe clinical forms of this disease are associated with homozygotic pattern of inheritance. In the family presented, the proband, even though heterozygous, he presented serious neurologic manifestations of FHBL. It is possible that this resulted not from a single abnormality but a combination of identified variants. 

The c.3696+1G>T mutation was identified by Walter at al. [13] as a loss-of-function variant responsible for altered triglyceride levels but at the moment of its identification in our study, it has not been registered in the Human Gene Mutation Database (HGMD), and there was no information on its clinical significance in the literature. Another variant found in the proband’s family, c.2188G>A, p.Val730Ile had been registered in the HGMD^®^ Professional 2018.2 mutation database [14]. This variant has been initially classified as a disease-causing mutation variant that is correlated with hypobetalipoproteinemia-induced nonalcoholic steatohepatitis symptoms. However, the same variant has been registered in the UniProt [15] and ClinVar [16] databases as a variant without clinical significance, or benign, or of uncertain significance. Its population frequency corresponds to 1.42% (1000G Project) [17] and 4.37% in the Polish healthy long-lived population (POLGENOM Project) [18], much above the estimated frequency of the disease (1 in 1000–3000 individuals) [5]. Based on these data, the c.2188G>A, p.(Val730Ile) variant should be considered to be a likely benign variant, according to the American College of Medical Genetics and Genomics (ACMG) recommendations [19]. In 2016, it was re-classified in the HGMD database as benign/likely benign. The next variant—c.8353A>C, p.(Asn2785His)—has been registered in ClinVar [16] as a benign variant. Its frequency in the general population corresponds to 1.18% in the 1000G Project and 0.4% in the POLGENOM Project [17,18]. The c.8353A>C, p.(Asn2785His) variant should also be considered as a benign variant, according to the ACMG recommendations [19]. 

Considering the novel c.3696+1G>T variant frequency in the general population (0.008% according to dbSNP built 148; present only as a single case in the National Heart, Lung and Blood Institute (NHLBI) Exome Sequencing Project cohort, rs141422999), no data on its occurrence in the Polish, long-lived, healthy cohort (POLGENOM Project); the results of bioinformatics analyses; and finally, the publication of Walter et al. [14], this variant should be considered pathogenic according to the ACMG recommendations.

Interestingly, another study identified a different single nucleotide variant at the same chromosomal position (c.3696+1G>C) affecting the donor splice sites of the intron 23 of the *APOB* gene. Functional studies revealed aberrant splicing of *APOB* RNA and the presence of a truncated apoB protein [20]. A direct sequencing of the *APOB* transcript isolated from CHOK1H8 cells expressing a minigene harboring the mutation showed that the c.3696+1G>C variant caused the formation of abnormal mRNA, retaining intron 23 and encoding a truncated apoB protein of 1276 amino acids [20].

These results support the idea that the splicing variant is the potential cause of the codominant FHBL. Identification of only one heterozygous pathogenic variant in the *APOB* gene in the proband (in one *APOB* allele) and the affected family members corresponded to the observed clinical symptoms of heterozygous FHBL (such as nonalcoholic fatty liver disease) and accounted for the late onset neurological complications. 

Based on the results of Cefalu et al. [20], we suggest that the truncated *APOB* protein produced in the proband, which is smaller than apoB-48 (the apoB form produced by the intestine and incorporated into chylomicrons), may lose the capacity to bind lipids and form chylomicrons secreted into the intestinal lymph and which may lead to inadequate intestinal lipid absorption. In heterozygous carriers of a truncating variant in *APOB*, fatty liver disease associated with low plasma levels of total cholesterol, was reported [9,20]. 

Neurologic disorders are well known in ABL. Serum apoB containing lipoproteins, very low (VLDLs) and low-density lipoproteins (LDLs) are absent, leading to a severe impairment of fat absorption and transport from the intestine resulting in very low serum triglycerides and cholesterol levels. In consequence, decreased absorption of vitamin E, as well as other vitamins and possibly other cofactors, are found. This leads to damage to the dorsal root ganglia, spinocerebellar tracts, retina, and cerebellum similar to those observed in vitamin E deficient mammals. Vitamin E is essential for neurological function and is transported in plasma in association with the apoB-containing lipoproteins [21]. The retinal degeneration, again peculiar to ABL, is thought to be caused by combined deficiency of vitamins A and E [22]. The decreased level or impaired function of apoB connected with the *APOB* gene mutation also lead to malnutrition and deficiencies in fat soluble vitamins (A and E). The consequence of vitamin E deficiency is demyelination of spinocerebellar axons, resulting in neurological symptoms, while demyelination of cranial nerves provokes ophthalmologic effects [6]. Additionally, the low level of vitamin A leads to abnormal function of retinal photoreceptors. 

In all our patients, including the proband, the serum levels of fat-soluble vitamins were normal, although near the low border of reference values which is typically stated in FHBL. Perhaps, however, long-term low concentrations of vitamins E and A, within the normal ranges, generate the development of neurological complications observed in our patients.

The severe neurological symptoms observed in the proband have not been described previously for FHBL heterozygotes, but this may be due to younger age of reported cases, as severe symptoms in our proband developed at the age of 70. Carriers of splicing mutations described by Cefalu at al. [20] were below the age of 50, while the compound heterozygotic proband who developed a mild and non-specific truncal hypotonia was only one month old. 

Neurological consequences, which are the major and life endangering complications of FHBL, emerge late in the course of the disease, but after development their progress is usually impossible to stop. 

Pattern electroretinogram (PERG) is a retinal biopotential evoked when a temporally modulated patterned stimulus of constant mean luminance is viewed. It may be altered in the macula or the retinal ganglion cells dysfunction, which do not significantly affect the a and b waves of the conventional full-field electroretinography (ERG). Thus, PERG receives clinical and research attention in both neurological and ophthalmological practice [23]. Clinically, PERG can be used in a patient with an abnormal visual evoked potentials (VEP) to establish whether a retinal (macular) disorder is present, and thus differentiate between macular and optic nerve dysfunction as a cause of VEP abnormality. It can also directly demonstrate retinal ganglion cell dysfunction. A flash ERG (FERG) is a test which measures the electrical response of the eye’s light-sensitive cells (rods and cones). It also checks other cell layers in the retina.

The delay of the P100 wave of pattern visual evoked potentials (PVEPs), although not a specific sign, is accepted in the diagnosis of the demyelination of the axons of the anterior part of the visual pathway [24]. In previous studies, delayed P100 latency was found in numerous diseases of the retina and visual pathway [25]. About 30% of analyzed cases were connected to retinal, mostly macular, diseases. 

In ERG, an abnormal implicit time suggests dysfunction of retinal cells and abnormal amplitude indicates their reduced number. A lower amplitude of the P100 wave and delayed latency found in family members with FHBL can be a consequence of abnormal retinal function primarily caused by vitamin A deficiency and/or impaired myelin formation due to inadequate lipid absorption. The pathology increases with age, which was confirmed by the differences in ERG recordings between family members who had preserved visual acuity and field. Buonuomo et al. described photophobia and polar cataract in one twin (9-year-old female with FBHL). Her twin brother had no ocular symptoms, but presented similar lowering of the Ganzfeld ERG amplitude [26]. Progressive degeneration of photoreceptors and other retinal cells leads to optic atrophy. In our opinion, PERG and FERG should be recommended in all patients with FHBL to detect the early signs of nutritional deficits and to monitor the progression of the disease. 

**Figure 8:**
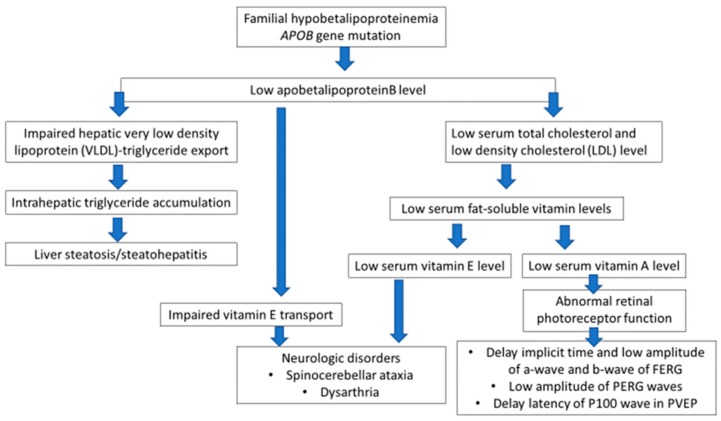
Schematic representation of clinical manifestations in familial hypobetalipoproteinemia (FHBL) as a result of *APOB* gene mutation (FERG—flash full-field electroretinography; PERG—transient pattern electroretinography; PVEP—transient pattern visual evoked potentials).

Only the proband complained of worsening vision, and that might be caused by initial senile cataract and abnormal retinal function in spite of full best-corrected visual acuity (BCVA). In cases of disturbed vision in elderly patients an ophthalmic examination is recommended to find atypical forms of retinitis pigmentosa [6]. As FBHL may lead to progressive demyelination, retinal degeneration, and even blindness, the monitoring of retinal function by ERG, combined with demyelination screening using VEP, could be helpful to allow early therapeutic decisions, to prevent ocular complications. The relationship between an *APOB* gene mutation and clinical manifestation of the FHBL is presented in Figure 8.

## 4. Materials and Methods 

Five relatives with FHBL were examined: the proband with long-lasting asymptomatic steatohepatitis who developed dysarthria and finally severe extrapyramidal syndrome with dysphagia and ataxia; his two children with liver steatosis/steatohepatitis; and two grandchildren. 

Written informed consent was obtained from all participants in this study. 

### 4.1. Genetic Testing

All of the genetic investigations performed in the proband and his family members were performed after informed consent. 

A peripheral blood sample was used as the source of DNA for testing, and DNA isolation was performed using the A&A DNA isolation kit (Blood Mini kit, A&A Biotechnology, Gdynia, Poland). A sequencing analysis of the whole coding region of the *APOB* gene, including 25 bp flanking intronic regions, was performed using the NGS method and the HaloPlex Design Panel (Agilent, Santa Clara, CA, USA), which also included LDL receptor (LDLR) gene enrichment probes. Sequencing was performed using the MiSeq Reagent Kit v2 (500 cycles) on a MiSeq^TM^ sequencer (Illumina, San Diego, CA, USA). 

For the *APOB* gene, the mean coverage corresponded to 842.68-fold; 98.79% of the bases were covered >50-fold, 0.71% of the bases were covered 10–49-fold, and 0.5% of the bases were covered 1–10-fold. There were no bases without any coverage. 

The presence of all variants was confirmed by direct DNA sequencing. Mutation Surveyor V 4.0.8 (Softgenetics, State College, PA, USA) was employed to analyze fluorograms. To describe variants, reference sequences according to HGMD^®^ Professional [13], gene names according to HUGO Gene Nomenclature Committee (HGNC), and variant names according to HGVS v15.11 were applied.

### 4.2. The Neurological Conditions 

The neurological conditions of the proband family members were evaluated using the Unified Parkinson’s Disease Rating Scale (UPDRS) and psychological tests. 

### 4.3. The Ophthalmological Examination 

The ophthalmological examination included BCVA and intraocular pressure measurement (Goldmann applanation tonometer, Harlow, UK); static perimetry (Octopus 1-2-3, glaucomatous program tG1; Interzeag, Schlieren, Switzerland); optical coherence tomography (OCT); and electrophysiological examinations, including transient PVEP, transient PERG, and FERG. Electrophysiological examinations were performed using Reti-Port (Roland Consult, Branderburg a.d. Havel, Germany). Examinations were conducted in compliance with the International Society for Clinical Electrophysiology of Vision (ISCEV) standards [27,28,29]. 

OCT to measure macula thickness and ganglion cell analysis were determined by Cirrus HD-OCT (Carl Zeiss, Oberkochen, Germany) after dilatation of 1% tropicamidum. Macular Cube 512 × 128 protocol was selected.

PVEP were stimulated by pattern reversal stimulations of 1° and 15′. Amplitude and latency of the P100 wave of PVEP, amplitude and implicit time of P50 and N95 waves of PERG, and amplitude and implicit time of a and b waves of FERG were measured. PERG and FERG were recorded with silver thread (DTL) electrodes.

## 5. Conclusions

In conclusion, the identified rare splicing variant c.3696 + 1G > T can be associated with autosomal, dominant FHBL with coexisting neurological symptoms. According to the literature, mutation databases, and population frequency, both additionally-identified missense variants could be excluded as the main cause of observed clinical signs. Electroretinography examination is a sensitive method for detection of early neuropathy, and should therefore be recommended for the care of patients with FHBL.

## Abbreviations

FHBL	Familial hypobetalipoproteinemia
APOB	Apolipoprotein B
NAFLD	Nonalcoholic fatty liver disease
ABL	Abetalipoproteinemia
NASH	Nonalcoholic steatohepatitis
MR	Magnetic resonance
CT	Computed tomography
TC	Total cholesterol
LDL	Low-density lipoprotein
TG	Triglycerides
DOB	Date of birth
NGS	Next-generation sequencing
HGVS	Human Genome Variation Society
OCT	Optical coherence tomography
PERG	Pattern electroretinography
PVEP	Pattern visual evoked potentials
ACMG	American College of Medical Genetics and Genomics
ERG	Electroretinography
LDLR	Low-density lipoprotein receptor
HGMD	Human Gene Mutation Database
UPDRS	Unified Parkinson’s Disease Rating Scale
BCVA	Best-corrected visual acuity
FERG	Flash full-field electroretinography
ISCEV	International Society for Clinical Electrophysiology of Vision
